# Wearable Enzymatic Alcohol Biosensor

**DOI:** 10.3390/s19102380

**Published:** 2019-05-24

**Authors:** Bob Lansdorp, William Ramsay, Rashad Hamid, Evan Strenk

**Affiliations:** Milo Sensors, Inc., California NanoSystems Institute (CNSI) Incubator, University of California Santa Barbara, Santa Barbara, CA 93106-6105, USA; will@milosensor.com (W.R.); rashad@milosensor.com (R.H.); evan@milosensor.com (E.S.)

**Keywords:** alcohol, biosensor, cartridge, disposable, transdermal, alcohol use disorder

## Abstract

Transdermal alcohol biosensors have the ability to detect the alcohol that emanates from the bloodstream and diffuses through the skin. However, previous biosensors have suffered from long-term fouling of the sensor element and drift in the resulting sensor readings over time. Here, we report a wearable alcohol sensor platform that solves the problem of sensor fouling by enabling drift-free signals in vivo for up to 24 h and an interchangeable cartridge connection that enables consecutive days of measurement. We demonstrate how alcohol oxidase enzyme and Prussian Blue can be combined to prevent baseline drift above 25 nA, enabling sensitive detection of transdermal alcohol. Laboratory characterization of the enzymatic alcohol sensor demonstrates that the sensor is mass-transfer-limited by a diffusion-limiting membrane of lower permeability than human skin and a linear sensor range between 0 mM and 50 mM. Further, we show continuous transdermal alcohol data recorded with a human subject for two consecutive days. The non-invasive sensor presented here is an objective alternative to the self-reports used commonly to quantify alcohol consumption in research studies.

## 1. Introduction

Every year, excess alcohol consumption in the United States is responsible for one in ten deaths of working-age people [1] and leads to $249 billion in economic costs, including $12 billion in specialty care for abuse/dependence [2]. Of the 18 million Americans with an Alcohol Use Disorder (AUD), only one in seven have received treatment, and those who have sought treatment have faced the daunting prospect of a relapse rate of between 20% and 80% [3]. Furthermore, all existing data on alcohol use in a treatment setting collected using self-report have been called into question by recent results [4], which demonstrated that 92% of patients drank during treatment, while fewer than half of patients reported drinking. Therefore, there is a need for new tools to better understand, diagnose, and treat AUD. A discreet and continuous wearable alcohol sensor would provide biophysical data that could be more reliable than self-reported consumption. Further, such a wearable technology could enable new treatments that connect patients in treatment for AUD to their clinicians and support network.

Continuous alcohol sensors have received growing interest from the research community [5,6,7,8,9,10,11,12,13,14], but commercial realizations thus far have involved trade-offs in performance [15,16]. The only two commercialized platforms SCRAM (Alcohol Monitoring Systems, Littleton, CO) and WrisTAS (Giner, Newton, MA) use platinum fuel cells. Baseline drift over time requires post-processing of the data [17], and possible degradation of the sensing element within days reduces data reliability [11,18,19]. Platinum fuel cells are further known to be prone to humidity-induced fouling [20,21]. Despite these drawbacks, efforts to introduce the SCRAM offender-monitoring bracelet into alcohol addiction treatment were encouraging, with a large majority (81%) of wearers reporting the bracelet to be useful in helping them reduce drinking, although social discomfort and physical irritation were observed, related to the physical shape and size of the bracelet [22,23].

In light of the limitations of existing wearable alcohol sensors and encouraging initial results in a treatment setting, what is desired is a non-invasive, continuous, and discreet alcohol sensor that overcomes the issue of sensor fouling and circumvents the limitations of self-reported alcohol consumption [8,24]. In this work, we describe a disposable cartridge system to overcome the problem of sensor fouling. We demonstrate how an enzymatic detection pathway enables a low detection limit. Finally, we miniaturize the entire system into a wearable device that can pair with a smartphone and demonstrate effective monitoring of a human subject’s transdermal alcohol concentration over a multi-day period.

## 2. Materials and Methods

### 2.1. Chemical Detection and Catalysis

**Figure 1:**
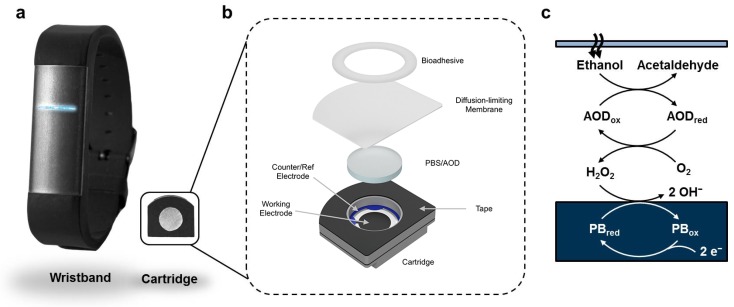
The primary functional components of the sensor: the wristband and disposable cartridge (**a**); a schematic depicting the primary functional components of the disposable enzymatic alcohol sensor cartridge (**b**); and the chemical pathway for detection within the sensor cartridge (**c**).

To convert alcohol that emanates from the skin into an electrical signal that can be digitized, we used the enzyme Alcohol Oxidase (AOD) (*Pichia pastoris*; Gwent) and a screen-printed Prussian Blue (PB) electrochemical sensor (DropSens) as the transducer. These key elements were contained within a custom-designed and manufactured disposable cartridge biosensor (Figure 1). Importantly, the disposable cartridge had a diffusion-limiting membrane (15 μm Polyethylene (PE) film; Goodfellow) that interfaced with the skin and had a surface area of 49 mm^2^, affixed to the cartridge by a closed-cell foam tape (TESA 75720) laser cut to shape. The cartridge reservoir (defined by a 1.59 mm thick die-cut low-density polyethylene piece) contained 1.3 units of AOD [25] in 25 μL of hydrogel consisting of 1× Phosphate-Buffered Saline (PBS) and agarose (MilliporeSigma) at pH 7.4. A 53 μL air-gap existed between the membrane and the hydrogel; ethanol traveled through the membrane, through the air-gap, and reacted with AOD to produce acetaldehyde with simultaneous formation of hydrogen peroxide. Hydrogen peroxide diffused to and was sensed by the electrode: a custom screen-printed PB [26,27,28,29] working electrode (surface area $21\;\mathrm{mm}{}^{2}$) and a Ag/AgCl quasi-reference electrode (surface area $6.0\;\mathrm{mm}{}^{2}$).

### 2.2. Laboratory Data Collection

In laboratory experiments, data were logged to a computer at 1 Hz with an NI-DAQ 6323 attached to a BNC-2090A (National Instruments), which accepted signals from a custom-built potentiostat configured to perform chronoamperometry at +93 mV with respect to the Ag/AgCl quasi-reference electrode, to exploit the non-linear selectivity towards hydrogen peroxide over oxygen [28]. Sensors were contained within a temperature-controlled chamber at 30 °C and measurements performed in triplicate. Syringe pumps were filled with either PBS solution or a PBS solution with ethanol of a known concentration, which was refreshed at 0.49 mL/h over the diffusion-limiting membrane to ensure a constant and known concentration of ethanol above the membrane.

### 2.3. Human Subject Data

**Figure 2:**
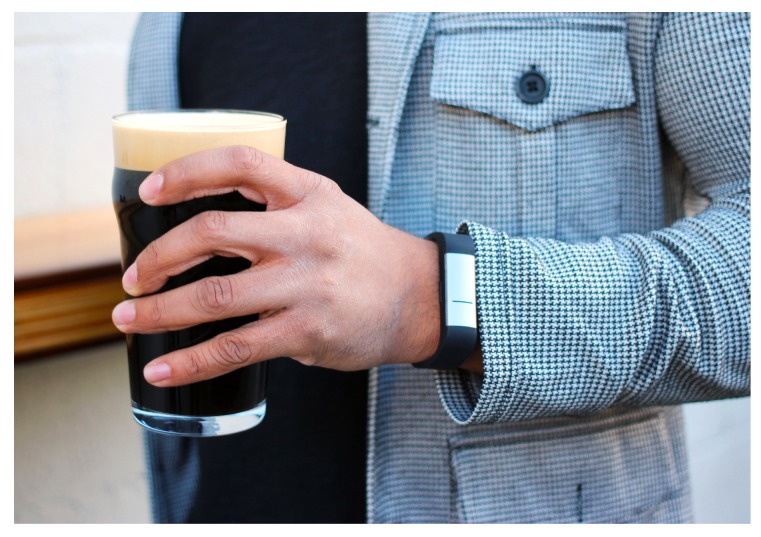
Photograph of the wearable alcohol sensor being worn in a real-world environment.

For human subject measurements, a miniaturized potentiostat [30,31] in a wearable wristband recorded electrical currents associated with the sensor and simultaneous temperature readings associated with a thermistor at 0.2 Hz with a 12-bit ADC. Values were transmitted to a corresponding smartphone application. The smartphone application sent data via a cellular antenna to a web server, where timestamps and electrical current were logged digitally for subsequent analysis. The electronics were enclosed in an injection-molded silicone wristband (see Figure 2), which was powered by a rechargeable lithium-polymer battery. The disposable cartridge was inserted into the skin-touching side of the wristband to make electrical contact with the miniaturized potentiostat circuitry via two gold-plated pogo pins on the wristband. The wristband was attached to the human subject via a tang buckle and worn in uniform contact with the dorsal side of the wrist.

The subject, the author B.L., wore a wristband with alcohol-sensing cartridges for two consecutive days on his left dorsal wrist. The subject carried one Apple iOS smartphone with a custom-designed app, which connected to the wristband via Bluetooth. Data from the smartphone were relayed to a server, where they were logged continuously. At t = 8.9 h, the subject consumed three 44 units of 35% alcohol in a period of 2 min and an additional 355 mL of 4.6% beer at t = 9.9 h. At t = 13.9 h, the subject went to sleep. Data were logged continuously up until an unexpected smartphone application disconnection was observed at t = 18.9 h, at which time, the app was restarted and data logging resumed. At t = 23.2 h, the wristband was removed from the wrist, the disposable cartridge was discarded, and the wristband was connected to a 5-V charger to recharge the lithium-polymer battery. At t = 25.7 h, a second new cartridge was inserted into the wristband, and data logging was resumed. Between t = 31.5 h and 31.8 h, the subject consumed three 44 units of 35% alcohol. At t = 40.6 h, another unexpected app crash was observed; however, the app was restarted, and collection resumed at t = 44.1 h. The study was concluded at t = 46.9 h.

It was impractical to collect breathalyzer data continuously, and therefore, we used the Widmark equation [32] (assuming 30 min of elimination time) to estimate peak BAC given the subject’s male sex, height of 1.97 m, mass of 90 kg, and mass of alcohol consumed for each respective day, yielding 0.086% on Day 1 and 0.061% on Day 2.

## 3. Results

### 3.1. In Situ Laboratory Results

**Figure 3:**
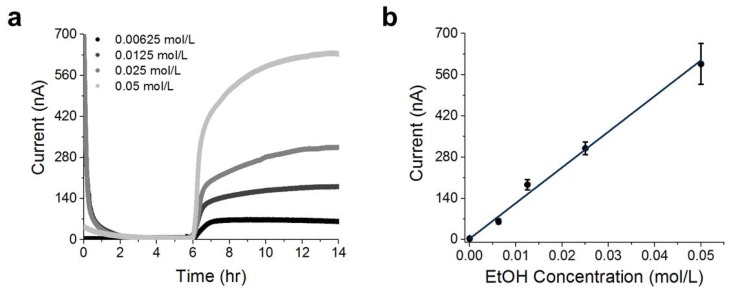
Laboratory characterization of the sensor response.

A disposable cartridge was initially connected via pogo pins to the recording electronics housed in a temperature-controlled chamber at 30 °C, and then, a 1× PBS solution was flowed over the diffusion-limiting membrane (Figure 3a). A spike in current was initially recorded, which was observed to decay to less than 50 nA within 48 min. The baseline current was recorded over a 6 h period, and a steady-state value of 6±3 nA was measured (t = 3–6 h). Then, a solution of 0.05 mol/L ethanol in 1× PBS was flowed over the diffusion-limiting membrane. Almost immediately, an exponential increase in current was observed, and a plateau current of 627±7 nA (t = 12–14 h) was recorded for over 8 h (representing a steady-state flux). Sensor response time (defined here as the time required for the current to reach 50% of the maximal plateau current after addition of a known concentration of ethanol) was measured to be 36±6 min with n = 12 sensors. The plateau currents were measured with different concentrations of ethanol, and we determined a linear sensor range of between 0 and 0.05 mol/L of ethanol (Figure 3b; error bars correspond to the standard deviation obtained from triplicate experiments at each concentration).

The fit proportionality constant between steady-state current and ethanol concentration was determined to be:

(1) $$m=\frac{208\;\mathrm{nA}}{0.0174\;\mathrm{mol}/L}$$

With a baseline current of less than 10 nA; a 208 nA signal represents a signal-to-noise ratio of over 20, without the use of any baseline current subtraction.

### 3.2. In Vivo Measurement Results

**Figure 4:**
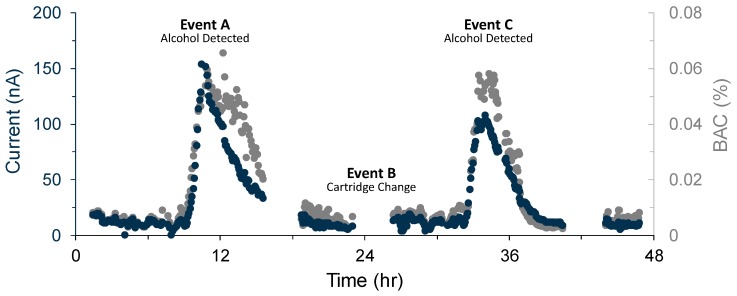
Electrical current measurements (blue) and Blood Alcohol Concentration (BAC) calculated using Equation (7) (gray) versus time.

The subject wore a wristband for two consecutive days, and the electrical current measured by two disposable sensors was plotted as a function of time (Figure 4). Data between t = 0 h and t = 1.5 h and between t = 25.5 h and 26.4 h correspond to “warm-up” periods when a new disposable cartridge was inserted, during which time current spikes occurred. These were removed for clarity of presentation. Data were median filtered over 60 s intervals.

## 4. Discussion

### 4.1. Transdermal Alcohol

There are two primary mechanisms by which alcohol emanates from the skin: passive diffusion (i.e., insensible perspiration) and active pumping through sweat glands (i.e., sensible perspiration) [33]. The physics of diffusion that govern the relationship between blood alcohol and insensible perspiration result in time-dependent kinetics [34,35]; transdermal alcohol is often described as being “delayed” with respect to blood alcohol concentration [36,37,38,39], although the relationship can be more accurately described as a convolution [17].

### 4.2. Membrane

Skin permeability to ethanol is known to fluctuate over time [40], and therefore, a flux-type sensor placed directly on the skin will measure the product of blood alcohol concentration and skin permeability, resulting in signals that vary with time [16]. The introduction of a diffusion-limiting membrane with known permeability converts a flux-type sensor into a continuous concentration sensor [41,42,43]. To enable robust alcohol measurement across a variety of skin types and sweating conditions, we introduced a membrane of well-defined permeability into our disposable cartridge design. The diffusion-limiting membrane limited the flux of ethanol to a membrane-limited rate when the wearer was sweating (sensible perspiration), while still enabling a detectable flux of alcohol when the wearer was not actively perspiring (insensible perspiration only).

Ethanol in the blood stream diffuses through an approximately 200 μm-thick epidermis layer, as well as the relatively impermeable, approximately 15 μm-thick, stratum corneum [35]. The second-order differential equations that govern blood to transdermal alcohol have been described previously [34], and we simplified them here by combining solubility in both blood $\beta_{b}$ and stratum corneum $\beta_{s}$, diffusion coefficient $D_{s}$, and the thickness of the stratum corneum $L_{s}$ into one permeability:

(2) $$k_{skin}\approx \frac{\beta_{s}D_{s}}{\beta_{b}L_{s}}=7.7\times 10^{-7}\;\mathrm{cm}/s$$

We added a diffusion-limiting membrane of $k_{membrane}$, and wrote net flux into the sensor *J* as the product of blood concentration $C_{Ethanol}$ and the reciprocal sum of permeabilities:

(3) $$J=C_{Ethanol}{\left(1/k_{skin}+1/k_{membrane}\right)}^{-1}$$

The permeability $k_{membrane}$ of the diffusion-limiting membrane was selected to be lower in permeability than the human skin $k_{skin}$, and thus, whether an individual is sweating (sensible perspiration with $k_{skin}>>k_{membrane}$) or not sweating (insensible perspiration only $k_{skin}>k_{membrane}$), the flux of alcohol into the sensor should theoretically be approximately:

(4) $$J\approx C_{Ethanol}k_{membrane}$$

We combined the flux calculation of Equation (4) together with the expected number of electrons generated per molecule of ethanol (assuming that downstream reaction kinetics were much faster than mass transport through the membrane), to write the expected electrical current as:

(5) $$i_{measured}=J\times F\times A\times N\times \eta +i_{background}=C_{Ethanol}\times k_{membrane}\times F\times A\times N\times \eta +i_{background}$$

For experimental values of $i_{measured}=597\pm 69\;\mathrm{nA}$ at a concentration of $C_{Ethanol}=0.05\;\mathrm{mol}/L$, with a surface area of the membrane $A=49\;\mathrm{mm}{}^{2}$, N=2 electrons/(molecule of EtOH), assuming η=100%, and with Faraday’s constant in terms of number of electrons of $F=9.6\times 10^{4}\;C/\mathrm{mol}$, solving yielded $k_{membrane}$ = $1.3\times 10^{-7}$ cm/s. As this result was less than the value of skin permeability to ethanol as previously published ($2.2\times 10^{-7}$ cm/s) [44] and as calculated in Equation (2) ($7.7\times 10^{-7}$ cm/s), we verified that the assumptions underlying Equation (4) were reasonable and our sensor was in a regime where transdermal fluxes of ethanol should be reliably measurable.

We experimentally verified our assumption that our steady-state sensor response was mass-transport-limited by the diffusion-limiting membrane (and not kinetically limited by PB or AOD), by measuring two sensors in identical conditions other than a factor of two difference in membrane thickness and observing the steady-state plateau currents to differ by a factor of two (Figure S1).

The electrical current was linearly proportional to alcohol concentration in Equation (5), and we deduced that the constant of proportionality was linear with membrane permeability. Of note, the expected electrical current was independent of enzyme and catalytic activity, although it was dependent on enzyme and catalyst efficiency.

We repeated plateau current measurements between 10 °C and 40 °C (Figure S2) and found that the steady-state plateau current followed an Arrhenius relationship:

(6) $$i\left(T\right)\propto e^{\frac{-E_{Pe}}{RT}}$$

where $E_{Pe}=103\;\mathrm{kJ}/\mathrm{mol}$, the ideal gas constant R=8.314 J/(mol·K), and T is the absolute temperature (K).

### 4.3. Connection and Steady State

When a disposable cartridge is first connected to the electronics, a spike in current is observed (Figure 3a), which we attributed to double-layer charging and cation diffusion through the PB [45,46,47]. We note that the four datasets were aligned by time of ethanol addition (see Supplemental Information for complete data sets). In the absence of alcohol, the current decayed to a steady-state value of approximately $i_{background}=6\pm 3\;\mathrm{nA}$. We performed measurements on a carbon electrode in the absence of the PB mediator (Dropsens DRP-110) (Figures S3 and S4), both with and without AOD, and these measurements demonstrated a −3 nA baseline current.

PB measurements at sufficiently low potential in the absence of hydrogen peroxide (Figure S5) were consistent with a previously-described oxygen signal [28], which asymptotically approached zero at higher applied potential. We concluded that our residual 6 nA baseline current was due to the non-zero reaction rate of PB with dissolved oxygen.

We attributed the low and stable baseline current firstly to the inherent specificity of PB towards hydrogen peroxide over other signals such as oxygen [28], in contrast to platinum fuel cells and other catalysts that report baseline signals up to 1000-times higher [48]. Secondly, the low drift can also be explained by the fact that our sensor was in a 100% humidity liquid environment and thus immune to drift in baseline current due to humidity changes that have plagued previous sensors [21].

### 4.4. Sensor Response

We measured the PB sensor response to hydrogen peroxide without AOD and in the absence of a membrane (Figure S5) and obtained a sensitivity of $24\;\mathrm{nA}{\mu M}^{-1}{\mathrm{cm}}^{-2}$ at 100 mV. This is over two orders of magnitude more than the fully-assembled cartridge sensitivity to ethanol given in Equation (1), further supporting our claim that PB reaction kinetics are not rate-limiting.

The sensor demonstrated a 36 min response time from baseline to 50% of plateau current (Figure 3), which may be limited by the diffusion time across the buffer layer $\tau \propto L^{2}/D$, a reservoir response time of τ∝V/m, the timescale associated with enzyme activity, or other sources. To rule out equilibration across the membrane as a significant contributor to response time, we measured two sensors with similar geometries, but with double the membrane thickness, and we observed response times within eight minutes of each other (Figure S1). Regardless of the physical origin of the sensor response time of 36 min at 30 °C, this is faster than a typical transdermal response time [39], and thus, further optimization would be of limited utility.

### 4.5. Enzyme in Excess

We varied the amount of added AOD in the sensor reservoir and observed little change to the plateau current above 1 U of added AOD (Figure S6), from which we conclude that the enzyme was in excess when there was greater than 1 U in the cartridge. This further supports our claim that steady-state sensor response given in Equation (1) is not kinetically limited by AOD or PB and is mass-transport limited [49] by the diffusion-limiting membrane.

In Figure S7, we measured ethanol with a cartridge containing only 0.1 U of AOD. By t = 6 h, the sensor plateau current began to decay towards zero. We expected that AOD in our sensor was degrading in agreement with previous measurements [50]. To investigate the origins of sensor plateau current decay, we removed the membrane of a cartridge after the decay event, then added additional ethanol, and found that it only responded again when new AOD was added. We thereby ruled out a change in membrane permeability, oxygen depletion in the reservoir, and concluded that enzyme deactivation was responsible for the finite cartridge lifetime. We speculate that our steady-state sensor response was robust to decreases in enzyme activity during its operational life, until enzyme activity had degraded to the point where the system switched from mass-transport limited across the membrane to kinetically limited by enzyme, at which time the measured electrical current began to decrease back to a baseline value.

### 4.6. Transdermal Measurements

Transdermal alcohol was measured continuously over two days (see Figure 4) to demonstrate a drift-free platform that overcomes sensor fouling. In periods where no alcohol signal was expected (t = 1.5 h to t = 8.9 h, t = 26.4 h to t = 31.5 h), the baseline current remained below 25 nA, matching the in vitro stability measured in Section 4.3. We have demonstrated two consecutive days of continuous measurement using our replaceable cartridge technology, and by induction, an infinite number of days can be detected consecutively without fouling by replacing the cartridge (Event B) on each successive day.

To convert the electrical current to BAC, we assumed that 0.08% was equivalent to 17.4 mM and that skin permeability was greater than our membrane as per Equation (4). We then used the laboratory-calibrated sensitivity in Equation (1), and added a temperature compensation term by taking the ratio of permeability at different temperatures, as described in Equation (6), to convert measured electrical current into an estimate of BAC(t):

(7) $$BAC\left(t\right)=i\left(t\right)\frac{0.08\%}{208\;\mathrm{nA}}e^{\frac{-E_{Pe}}{R}\left(\frac{1}{T_{0}}-\frac{1}{T(t)}\right)}$$

where $T_{0}=303\;K$ is the temperature at which the laboratory sensitivity calibration was performed and T(t) is the time-dependent temperature as read by a thermistor in the wristband. We plot the BAC estimates together with measured electrical current in Figure 4.

On Day 1 (0–24 h), the electrical current was observed to rise significantly above the baseline current (> 50 nA), at t = 10.10 h, or 70 min after alcohol was consumed (Event A). Based on the weight of the subject and the alcohol consumed, we calculated a theoretical peak BAC of 0.086%, and measured a peak BAC of 0.059% (ignoring a short-lived and biologically-unrealistic outlier at t = 12.4 h). On Day 2 (24–48 h), the current was again observed to rise significantly above the 50 nA baseline current at t = 33.0 h, or 90 min after alcohol consumption (Event C). We calculated a theoretical peak BAC of 0.061% and recorded a peak BAC of 0.058%. Compared to our predictions of BAC, our sensor was slightly underestimating BAC. This could be due to the influence of skin acting as a reservoir for ethanol and a diffusive barrier. Continuous measurements of skin hydration state could perhaps partially compensate for this effect and improve on Equation (7). The response times were similar to previously-published values for transdermal alcohol [39]. We note that our sensor was able to cover the subject’s descending BAC, while the subject was sleeping, a feat impossible with commonly-used self-report surveys [51].

Future work could improve on app and Bluetooth connection reliability. The gap caused by charging the wristband between study days could be easily overcome in clinical research by having two wristbands per participant, one charging while the other is being worn.

An assumption of this work is that the diffusion-limiting membrane is lower in permeability to ethanol than the skin. If this assumption is violated, the measured signal would be lower than the prediction of Equation (4). Future work to measure skin permeability $k_{skin}$ and its effect on sensor response in a wide range of environmental conditions would be of interest, particularly in a range of temperatures and skin sweating conditions. We speculate that the on-skin response time could be faster in the presence of sensible perspiration such as during physical exertion or elevated temperature.

## 5. Conclusions

We have demonstrated transdermal alcohol measurement over two consecutive days by using a miniaturized biosensor that mates with a discreet wristband, highlighting how a disposable cartridge can overcome the issue of sensor fouling that plagued previous transdermal alcohol sensors. Measurements with a human subject demonstrated the ability to capture real-world drinking events, with an increase in signal corresponding to alcohol consumption and a decrease back to baseline corresponding to a return to sobriety while sleeping. The wearable described in this work has broad implications for the field of alcohol research, potentially enabling a new generation of researchers to perform non-invasive measurements in the field to improve on self-reported alcohol consumption. We expect that the transdermal alcohol sensing technology described above will find immediate application in research studies that seek to replace self-reported alcohol consumption with physiological data and may find more broad commercial uses in the future.

## 6. Patents

We have patented the work in this manuscript: [52].

## Supplementary Materials

The following are available online at https://www.mdpi.com/1424-8220/19/10/2380/s1: Figures S1–S7 and raw data files used to generate Figure 3 and Figure 4.
