# Supplementary material for: Wearable Enzymatic Alcohol Biosensor
Source: Sensors (Basel). 2019 May 24;19(10):2380. doi: 10.3390/s19102380 (PMC6566815; doi:10.3390/s19102380)
Supplement: Supplementary file 1 [file sensors-19-02380-s001.zip › Supplementary_Information/Wearable_enzymatic_alcohol_bio_sensor_Supplemental.pdf]

Supplementary Materials: Wearable enzymatic alcohol biosensor

Bob Lansdorp*, William Ramsay, Rashad Hamid, and Evan Strenk

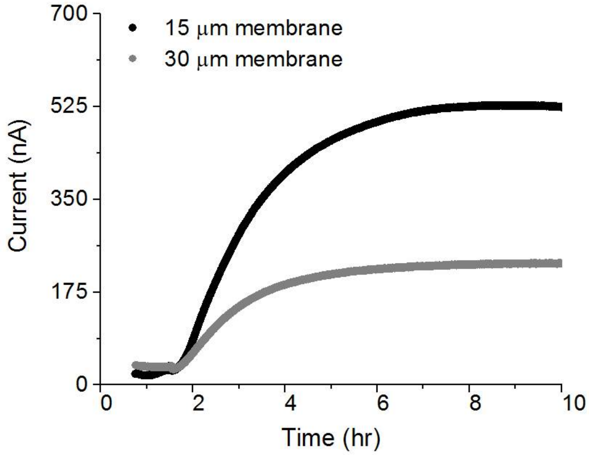

Figure S1. Amperometric measurement of two cartridges, assembled identically, but with with two different diffusion-limiting membranes from the same manufacturer (Goodfellow 15 µm thickness and Goodfellow 30 µm thickness), with measured response times of 77 min and 69 min, respectively. Cartridge volume was 78 µL

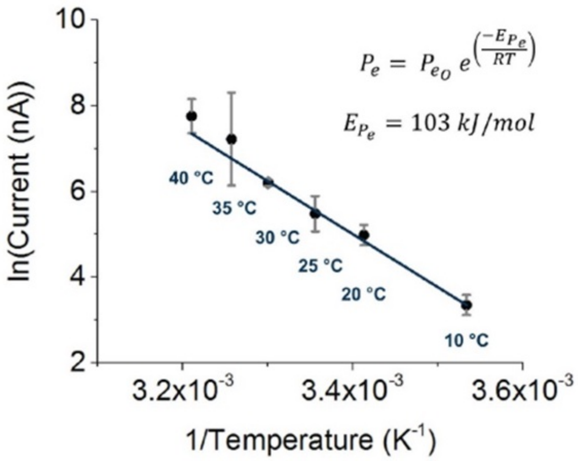

Figure S2. Plateau current measurements from amperometric experiments with constant 50 mM Ethanol concentration. Temperature was varied between 10 - 40 °C using a thermoelectric incubator. Error bars correspond to the standard deviation obtained from experiments at each temperature. Temperature dependence of permeability is fit to an Arrhenius function [1].

References

1. Flaconneche, B.; Martin, J.; Klopffer, M. Permeability, diffusion and solubility of gases in polyethylene, polyamide 11 and poly (vinylidene fluoride). *Oil & Gas Science and Technology* **2001**, *56*, 261–278.

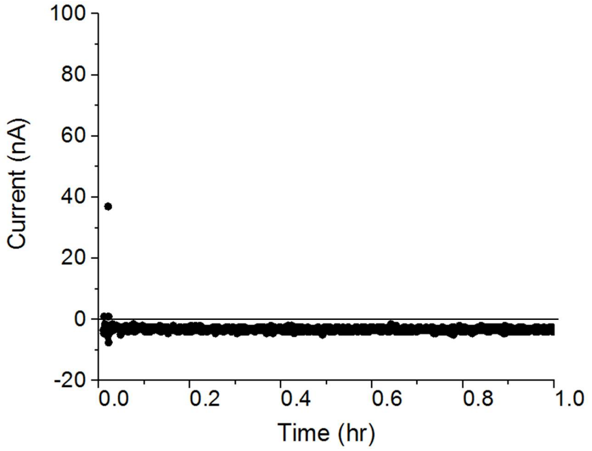

Figure S3. Amperometric measurement of a carbon electrode without mediator in 1x PBS solution at +93mV.

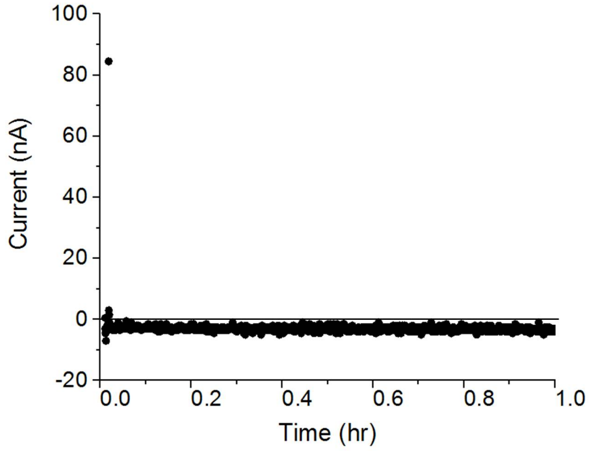

Figure S4. Amperometric measurement with added AOD enzyme in 1X PBS, performed at +93mV.

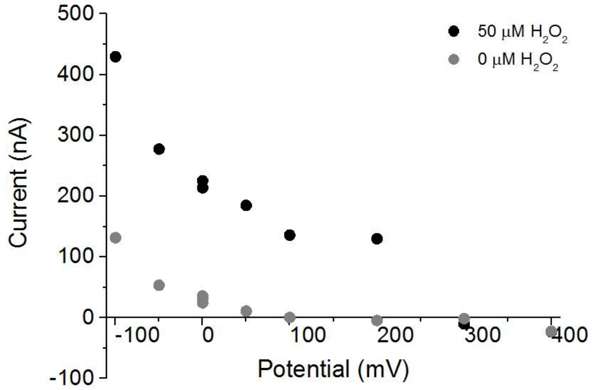

Figure S5. Amperometric measurements of plateau currents, before and after addition of 50 μM hydrogen peroxide. Measurements were performed at various applied potentials in a stirred beaker with 10 mL PBS solution. Measurements were performed in a three-electrode setup with potential controlled with respect to a saturated Ag/AgCl reference electrode (CH Instruments). Note that the quasi-reference electrode of the sensor in the main text was measured using chronopotentiometry to be +20mV relative to saturated Ag/AgCl reference electrode data shown above.

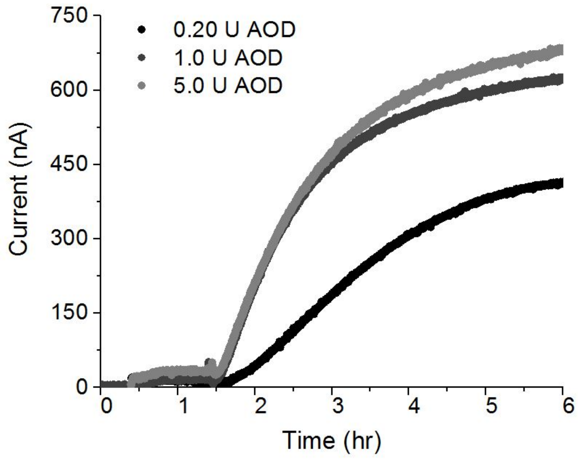

Figure S6. Amperometric response of the sensor to the addition of 50 mM ethanol, flowed at 0.49 mL/min with a syringe-pump setup over the membrane. Measurements were performed with varying amounts of AOD in the reservoir (0.2U, 1.0U, 5.0U).

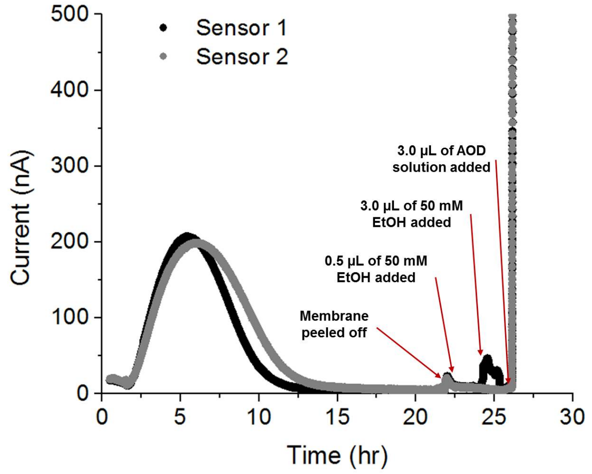

Figure S7. Amperometric measurements of two biosensors (0.1U of AOD added initially) to a constant 50 mM of ethanol flowed over sensor membrane. By t=15 h, current degraded to 0 nA. Exposure to ambient air (t=22hr) and addition of more ethanol (t=25hr) had little effect on current. Upon addition of fresh enzyme to a cartridge (t=26hr), resurgence in measured current is observed.
