# Supplementary figures and images for: Wearable Enzymatic Alcohol Biosensor
Source: Sensors (Basel). 2019 May 24;19(10):2380. doi: 10.3390/s19102380 (PMC6566815; doi:10.3390/s19102380)

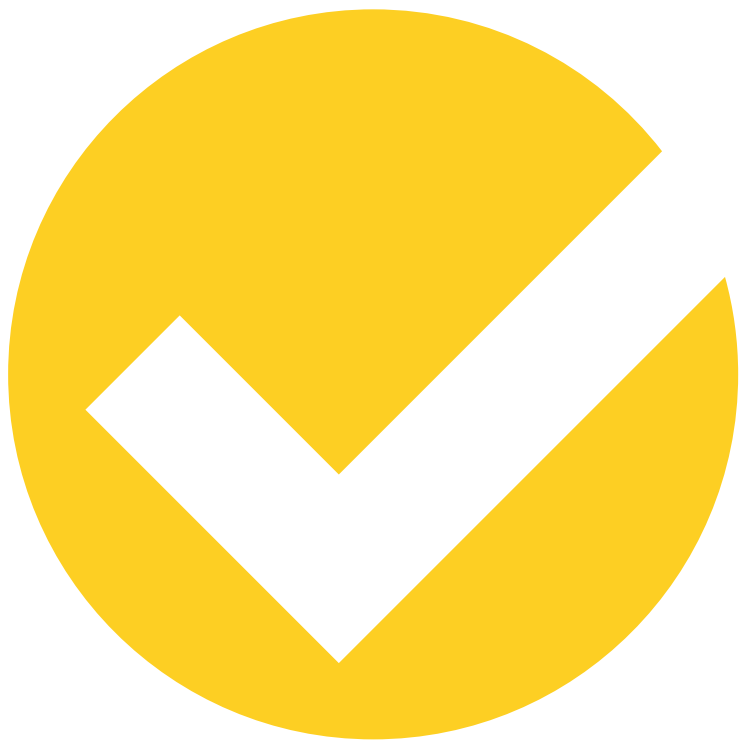

check for  
updates

Supplement: Supplementary file 1 [file sensors-19-02380-s001.zip › Supplementary_Information/Definitions/logo-updates.pdf]

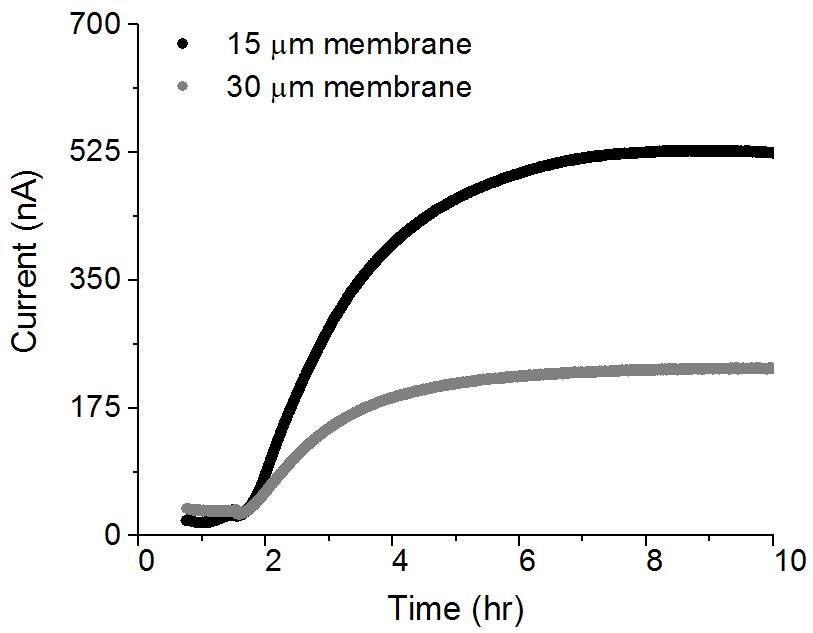

Supplement: Supplementary file 1 [file sensors-19-02380-s001.zip › Supplementary_Information/SupFigs/FigureS1_v2_t2t4.jpg]

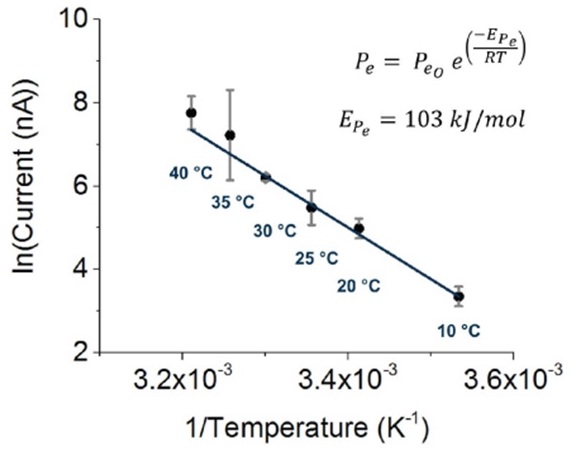

Supplement: Supplementary file 1 [file sensors-19-02380-s001.zip › Supplementary_Information/SupFigs/FigureS2_v2_temp.jpg]

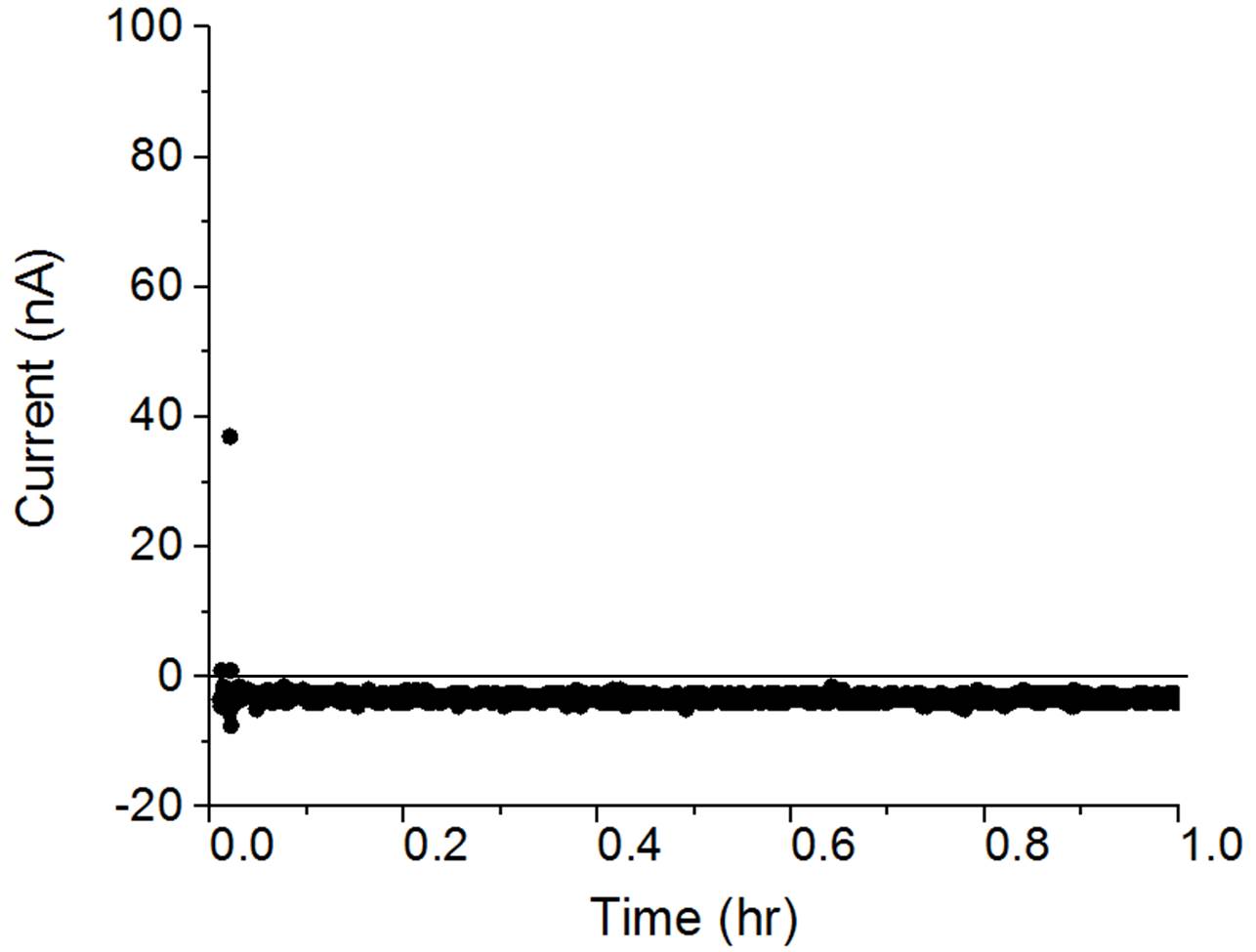

Supplement: Supplementary file 1 [file sensors-19-02380-s001.zip › Supplementary_Information/SupFigs/FigureS3_v2_110noAOD.jpg]

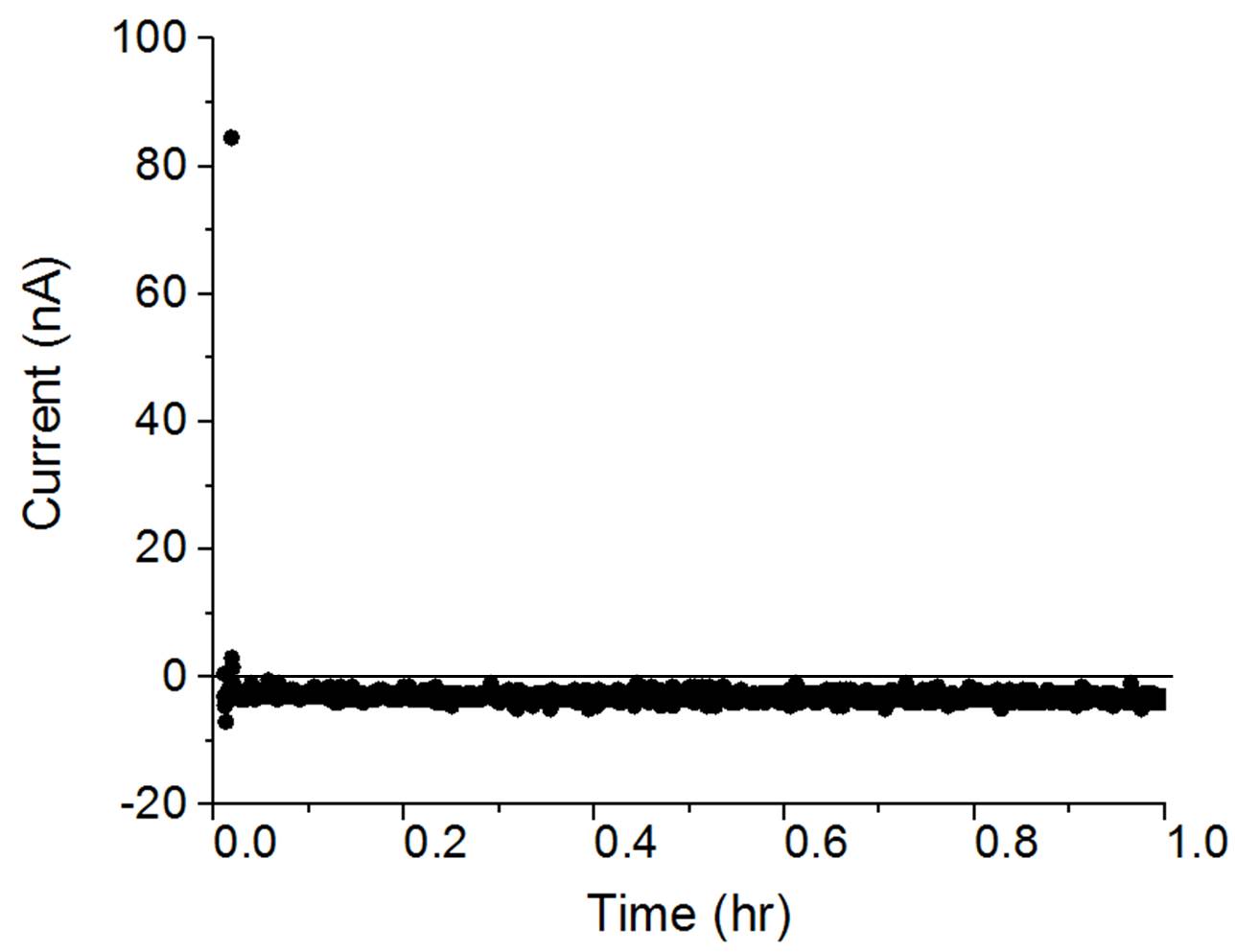

Supplement: Supplementary file 1 [file sensors-19-02380-s001.zip › Supplementary_Information/SupFigs/FigureS4_v2_110AOD.jpg]

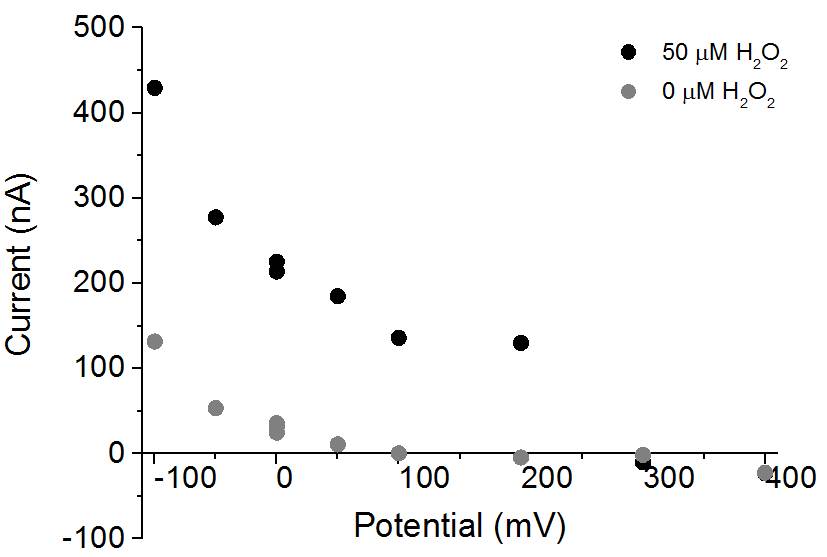

Supplement: Supplementary file 1 [file sensors-19-02380-s001.zip › Supplementary_Information/SupFigs/FigureS5_v2_potential.jpg]

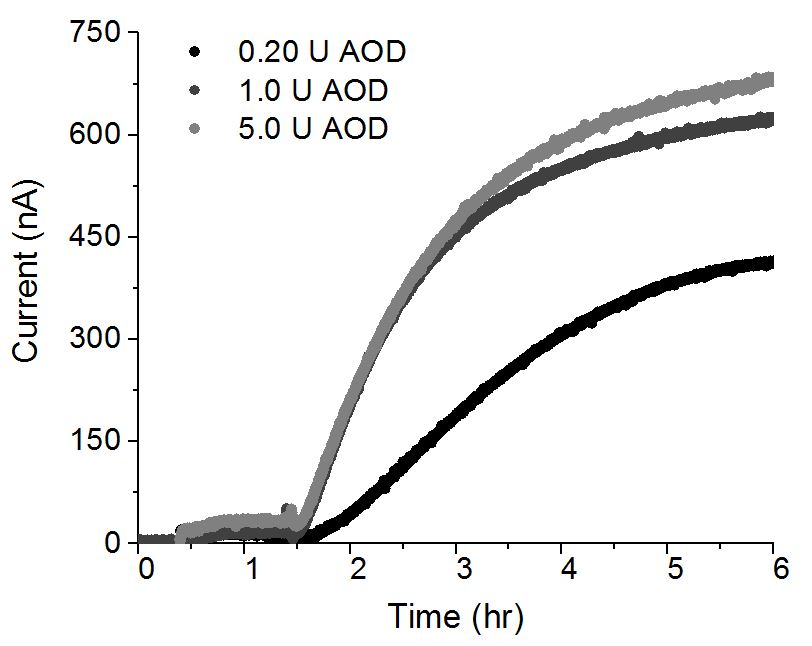

Supplement: Supplementary file 1 [file sensors-19-02380-s001.zip › Supplementary_Information/SupFigs/FigureS6_v1_AODexcess.jpg]

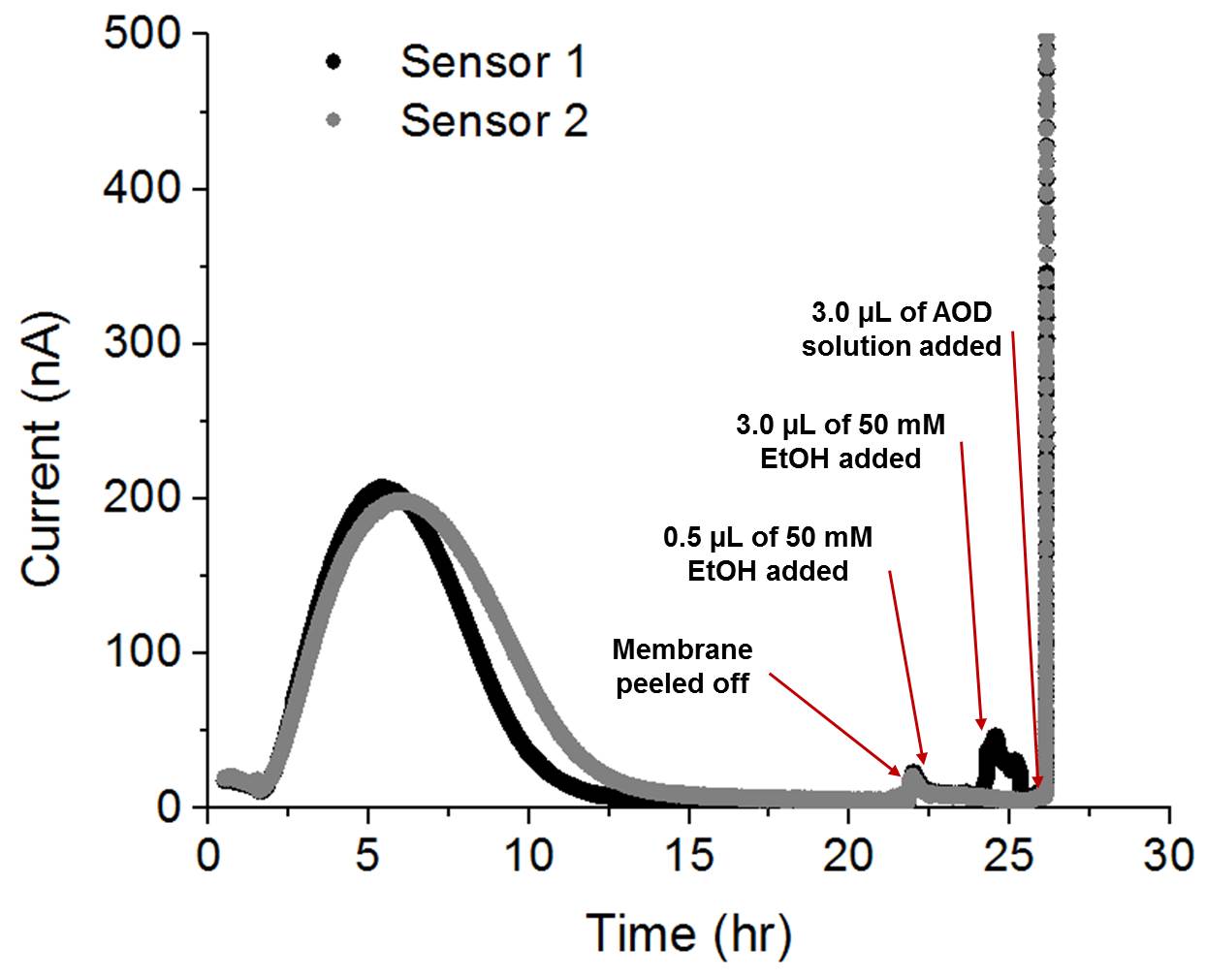

Supplement: Supplementary file 1 [file sensors-19-02380-s001.zip › Supplementary_Information/SupFigs/FigureS7_v1_cartridgedeath.jpg]
